# Origin and Characterization of Extracellular Vesicles Present in the Spider Venom of *Ornithoctonus hainana*

**DOI:** 10.3390/toxins13080579

**Published:** 2021-08-20

**Authors:** Chengfeng Xun, Lu Wang, Hailin Yang, Zixuan Xiao, Min Deng, Rongfang Xu, Xi Zhou, Ping Chen, Zhonghua Liu

**Affiliations:** The National and Local Joint Engineering Laboratory of Animal Peptide Drug Development, College of Life Sciences, Hunan Normal University, Changsha 410081, China; xuncf@hunnu.edu.cn (C.X.); WangluLLWW@163.com (L.W.); YANGhailinyang@163.com (H.Y.); xzxuaner@163.com (Z.X.); dengmin0714@163.com (M.D.); xurongfang1996@outlook.com (R.X.); xizh@hunnu.edu.cn (X.Z.)

**Keywords:** *Ornithoctonus hainana*, spider, venom, extracellular vesicles

## Abstract

Extracellular vesicles (EVs), including exosomes and microvesicles, are membranous vesicles released from nearly all cellular types. They contain various bioactive molecules, and their molecular composition varies depending on their cellular origin. As research into venomous animals has progressed, EVs have been discovered in the venom of snakes and parasitic wasps. Although vesicle secretion in spider venom glands has been observed, these secretory vesicles’ origin and biological properties are unknown. In this study, the origin of the EVs from *Ornithoctonus hainana* venom was observed using transmission electron microscopy (TEM). The *Ornithoctonus hainana* venom extracellular vesicles (HN-EVs) were isolated and purified by density gradient centrifugation. HN-EVs possess classic membranous vesicles with a size distribution ranging from 50 to 150 nm and express the arthropod EV marker Tsp29Fb. The LC-MS/MS analysis identified a total of 150 proteins, which were divided into three groups according to their potential function: conservative vesicle transport-related proteins, virulence-related proteins, and other proteins of unknown function. Functionally, HN-EVs have hyaluronidase activity and inhibit the proliferation of human umbilical vein endothelial cells (HUVECs) by affecting the cytoskeleton and cell cycle. Overall, this study investigates the biological characteristics of HN-EVs for the first time and sheds new light on the envenomation process of spider venom.

## 1. Introduction

Spiders (Araneae) are the most species-rich terrestrial arthropods after insects. Due to their adaptive physiological structure and diversified ecological behaviors, they are widely distributed in almost all terrestrial ecosystems [1]. Venom glands are specialized organs formed during the evolution of spiders, and the secreted venom is a powerful tool for spiders to capture prey and for defense [2]. The major spider venom component groups are small molecular mass compounds, toxin peptides (cytolytic and neurotoxic), enzymes, and proteins [3]. These molecules, with different biological activities, are mixed in the venom, resulting in a multi-strategic weapon for spiders. The natural biologically active molecules in venom provide a vast “treasury” for disease research, drug development, and pest control [4,5,6].

Recent research has shown that cells can communicate “long-distance” or even cross-species via various types of membrane particles known as extracellular vesicles (EVs) [7,8]. These nano-sized vesicles, abundant in biological fluids, can selectively wrap various proteins, lipids, and nucleic acids from secreted cells and are widely involved in transmitting information between cells and regulating various physiological processes [9]. In lower organisms, the release or secretion of EVs is a common cellular mechanism. EVs are found in archaea, bacteria, and parasites, which may be due to the evolutionary existence of a common ancestor [10,11,12]. For arthropods, EVs are also involved in the physiological activities and life histories of fruit flies, mosquitoes, and ticks [13,14,15].

EVs have been discovered in snake venom and parasitic wasp venom. However, the EVs reported in the venom of venomous animals are known by different names, including microvesicles (*Crotalus durissus terrificus*) [16], exosome-like vesicles (*Gloydius blomhoffi blomhoffi*) [17], small membranous vesicles (*Gloydius blomhoffi blomhoffi*) [18], mixed-strategy extracellular vesicles (*Leptopilina* *heterotoma*) [19], venosomes (*Leptopilina* *heterotoma* *and* *Leptopilina* *boulardi*) [20], and extracellular vesicles (*Leptopilina* *heterotoma*) [21]. The functional molecules in the EVs of snake (*Agkistrodon contortrix*) venom are primarily virulence-related proteins, with significant cytotoxic activity [22]. Parasitoid wasp venom EVs contain immunosuppressive proteins that can target and kill the *Drosophila’s* lamellocyte, increasing the success of wasp parasitism on certain *Drosophila* species [19,20]. The above research indicates that the practice of venomous animals secreting EVs in their venom seems to be a conservative and common phenomenon. The EVs in the venom contain newly synthesized venom components and show related biological activity. On the other hand, the EVs’ flexible envelope structure may assist in the mixed transportation of venom components during the envenomation process.

To date, although vesicle secretion has been observed in spider venom glands [23,24,25,26,27], the biological characteristics of these secretory EVs are still unknown. Therefore, we used morphological analysis and LC-MS/MS–based proteomics strategies to investigate the origin and characteristics of the *Ornithoctonus hainana* venom extracellular vesicles (HN-EVs). For the first time, we isolated EVs from *Ornithoctonus hainana* venom and analyzed their morphology and protein cargo composition. Finally, the hyaluronidase activity of HN-EVs and their cytotoxicity to HUVECs were verified and evaluated. These observations further confirm the presence of EVs in *Ornithoctonus hainana* venom and provide new insights into the spider envenomation process.

## 2. Results

### 2.1. Ultrastructural Analysis of the Glandular Epithelium Cells of the Ornithoctonus hainana Venom Gland by TEM

**Figure 1:**
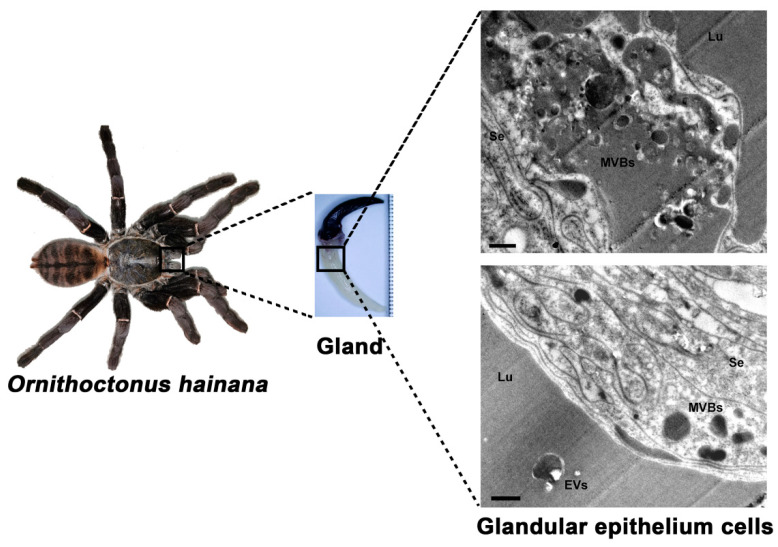
Ultrastructure of glandular epithelium cells from *Ornithoctonus hainana* venom gland. The adult *Ornithoctonus hainana* is about 15 cm long (leg diameter), with a 2 cm venom gland. Transmission electron microscopy was used to observe a cross-sectional view of the gland and the secretion of EVs by glandular epithelium cells. Secretory epithelium (Se), lumen (Lu), extracellular vesicles (EVs), multi-vesicular bodies (MVBs). Scale bar: 100 nm.

The adult *Ornithoctonus hainana* has a body length (leg diameter) of about 15 cm and a venom gland length of about 2 cm (Figure 1). The venom glands of *Ornithoctonus hainana* were cross-sectioned and observed by transmission electron microscopy. Many secretory vesicles contained high-density substances at the edge of the glandular epithelium cell membrane of the spider venom glands, showing a typical multi-vesicular body (MVB) structure. An apparent extracellular vesicle (EV) structure was observed in the venom gland lumen (Lu) (Figure 1). The findings indicate that EVs are secreted in the venom glands of *Ornithoctonus hainana,* and the origin of HN-EVs seems to be consistent with exosomal biogenesis [28].

### 2.2. Isolation and Characterization of Secreted EVs from the Venom of Ornithoctonus hainana

**Figure 2:**
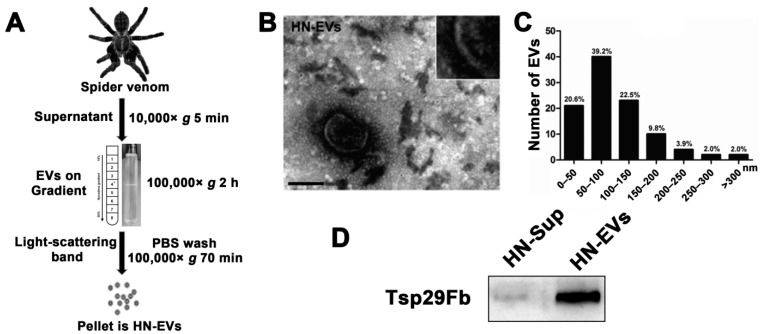
Isolation and characterization of secreted EVs from the venom of *Ornithoctonus hainana.* (**A**) Schematic of the HN-EVs isolation protocol from spider venom. Asterisk mark near the middle of the centrifuge tube with a Nycodenz gradient indicates level of the light-scattering band. (**B**) TEM analysis showed that the purified HN-EVs have a classic exosomal cup-shaped and lipid bilayer membrane morphology. Scale bar: 100 nm. (**C**) Statistical analysis of the particle size distribution of HN-EVs in TEM images shows that the particle size of HN-EVs is mainly distributed between 50 and 150 nm (61.7%) (*n* = 102). (**D**) Western blotting detected the expression of arthropod EV marker Tsp29Fb in HN-EVs. *Ornithoctonus hainana* venom extracellular vesicles (HN-EVs), venom centrifugal supernatant (HN-Sup).

After observing the apparent MVB structure and vesicle secretion in the glandular epithelium cells, we further isolated and identified the vesicles secreted in spider venom. We developed a method for isolating EVs from spider venom (Figure 2A) based on the research of parasitic wasp venom EVs [19]. Furthermore, the isolated EVs’ electron microscopy morphology, particle size morphology, and marker expression were characterized. The light-scattering band (asterisk) precipitate collected by density gradient centrifugation contained membranous vesicles of about 100 nm (Figure 2A,B). The TEM image structure of HN-EVs was similar to that of mammalian EVs [29] and snake venom EVs [18,22]. HN-EVs’ structure was complete, with a typical exosomal cup-shaped and lipid bilayer membrane (Figure 2B). According to the EV results (*n* = 102), the diameters of the EV particles in the venom were mainly distributed between 50 and 150 nm (Figure 2C). We further verified the expression of the arthropod EV marker protein Tsp29Fb (homologous to the mammalian exosomal marker CD63) [14] in HN-EVs (Figure 2D). The above results indicate that EVs are present in spider venom and their characteristics are consistent with those of typical extracellular vesicles.

### 2.3. Analysis and Identification of HN-EV Proteins

**Figure 3:**
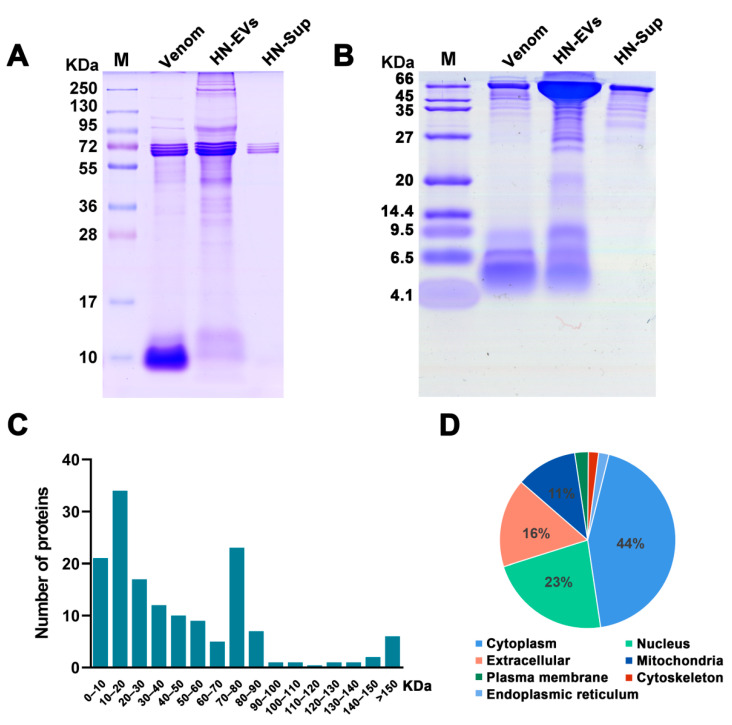
Analysis and identification of HN-EV proteins. (**A**) SDS-PAGE analysis of HN-EVs, venom, and HN-Sup. (**B**) Tricine-PAGE analysis of HN-EVs, venom, and HN-Sup. (**C**) Evaluation of molecular weight distribution of 150 proteins identified by LC-MS/MS. (**D**) The cellular component analysis showed that HN-EV proteins were found mainly in the cytoplasm, extracellular, nucleus, and mitochondria.

To visualize the protein content of HN-EVs, SDS-PAGE analysis was performed and compared with the whole venom and centrifugal supernatant. With the same amount of protein loaded, the whole venom was shown to be rich in small molecule proteins (<15 KDa). In comparison, HN-EVs were rich in high molecular weight (>95 KDa) proteins (Figure 3A), which suggests that HN-EVs may contain more high molecular weight membrane proteins and that the whole venom contains more peptides. Bands of around 72 kDa in the centrifugal supernatant (HN-Sup) of the maximum loading volume (30 µL) may indicate an abundance of hemocyanin in the venom. To evaluate the difference of smaller molecular proteins, further analysis by Tricine-PAGE was performed and showed HN-EVs to be largely in the molecular weight range of 9.5–35 kDa (Figure 3B). It has been shown that venom components can be transported into the gland lumen via EVs [27]. The protein profile of EVs in our study appears to be similar to that of the whole venom, though with different molecular weight distributions. Thus, our observations further support the previous finding that venom components can be secreted into the gland lumen via EV transportation.

To identify protein composition, HN-EVs were analyzed by the shotgun proteomics method based on LC-MS/MS. A total of 483 high-confidence peptides corresponding to 150 non-redundant proteins were identified in HN-EVs (Supplementary Tables S1 and S2). The proteins identified in this study were mainly distributed between 10 and 90 KDa; this result is consistent with the protein profile analyzed by gel electrophoresis (Figure 3A,C). The cellular component analysis showed that HN-EV proteins were found mainly in the cytoplasm (44%), extracellular (23%), nucleus (16%), mitochondria (11%), plasma membrane (3%), cytoskeleton (2%), and endoplasmic reticulum (1%) (Figure 3D).

### 2.4. Functional Classification of HN-EV Proteins

**Figure 4:**
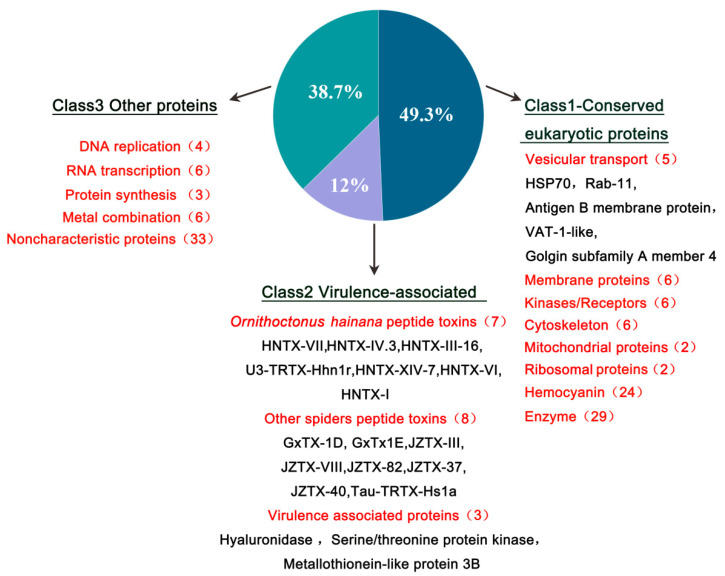
Functional classification of HN-EV proteins. After a database search, 150 proteins were identified with high confidence. Select HN-EV proteins, many of which are expected to be vesicular transport-associated and virulence-associated proteins, are displayed by their proteomic classes (1–3; bulleted within descriptive subclasses).

Our previous transcriptomic, peptidomic, and genomic research revealed the molecular diversity of the venom of *Ornithoctonus hainana* [30,31,32]. These studies show how venom’s complex components interact to produce a wide range of bioactivities that aid in adaptation to changing environments and prey types. Therefore, to better understand and assess the potential functions of HN-EVs, we divided the identified proteins into three classifications (Figure 4): conservative eukaryotic proteins (49.3%; Class 1), virulence-related peptides/proteins (12%; Class 2), and other proteins with potential functions (38.7%; Class 3).

Class 1 consists of conservative eukaryotic proteins containing vesicle conservative transport-related proteins and membrane proteins (Table 1). HSP70 and Rab-11 are proteins associated with vesicle transport, found in parasitic wasp venom EVs [19]. HSP70 is a marker of mammalian EVs [33] and is highly expressed in arthropod (mosquitoes and ticks) EVs [14,34]. Rab11 is a calcium-binding protein that participates in intracellular vesicle transport by binding to mammalian myosin-5b [35]. Other proteins potentially related to vesicle transport include the golgin subfamily A member 4 protein, synaptic vesicle membrane VAT-1-like protein, and putative antigen B membrane protein. The identification of these proteins further verified the vesicle-related origins of EVs in the venom of *Ornithoctonus hainana*.

The virulence-related peptides/proteins (Class 2) include peptide toxins, hyaluronidase, serine/threonine protein kinase-like proteins, and metallothionein-like proteins (Table 1). We identified 15 toxin-like peptides, the majority of which are neurotoxic peptides, from our previously published data library [30,36]. Among them, *Ornithoctonus hainana* toxins include HNTX-VII, HNTX-IV.3, HNTX-III-16, U3-TRTX-Hhn1r, HNTX-XIV-7, HNTX-VI, and HNTX-I. These toxins primarily affect the sodium channels of target cells and have the ability to paralyze target species [31]. The other spider peptide toxins discovered may be enzymatically digested peptides that match species homologous peptide toxins, such as Tau-TRTX-Hs1a, GxTX-1D, GxTx1E, JZTX-III K, JZTX-VIII, JZTX-82, JZTX-37, and JZTX-40. This also suggests that these spider venoms are related in evolutionary terms. Hyaluronidase is an important component of spider venom and a diffusion factor for toxic compounds [37]. These results indicate that there is a mixture of multiple virulence factors in HN-EVs. Two other potential virulence-related proteins, serine/threonine kinase-like protein and metallothionein-like protein, corresponding to serine proteases and metalloproteinases, respectively, may also play a role in the envenomation process [38,39,40], but their functions still need to be further verified. Class 3 includes replication, transcription-related element proteins, protein synthesis, metal combination, and proteins with unknown functions. These “non-toxin” components found in HN-EVs suggest that the vesicle-origin pathway may be the primary source of non-toxic components in venom.

### 2.5. Validation of Hyaluronidase Activity

**Figure 5:**
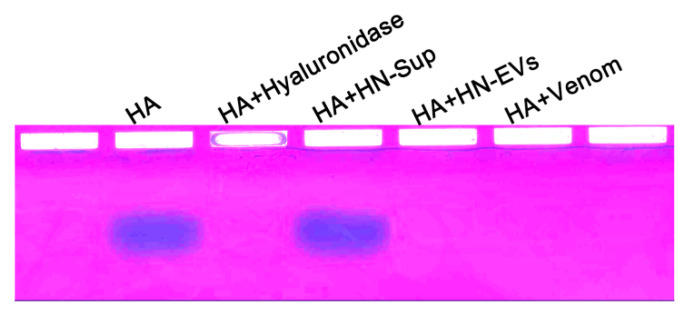
Hyaluronidase activity of HN-EVs. Hyaluronic acid (HA) was incubated with HN-EVs, HN-Sup, venom, and hyaluronidase at 40 °C overnight and separated on a 1% agarose gel at 50 mA for 3 h. Degradation was revealed by sequential toluidine blue and Stains-All staining.

Theraphosid spider venoms frequently include hyaluronidase-like enzymes [37]. LC-MS/MS analysis found virulence-related hyaluronidase in HN-EVs. To evaluate if HN-EV functionally resembles whole venom activity, the hydrolysis activity of HN-EV hyaluronate was measured. HN-EVs and whole venom were mixed with hyaluronic acid at a ratio of 4:1 (*w*/*w*), incubated at 40 °C for 12 h, and then subjected to electrophoresis and staining analysis. HN-EVs and whole venom were consistent with the positive control (hyaluronidase), reducing the molecular weight of hyaluronic acid and making it non-staining. Hyaluronic acid was not hydrolyzed in the negative control (centrifugal supernatant) (Figure 5). This indicates that HN-EVs and whole venom have hyaluronidase activity. The co-injection of hyaluronidase and the spider toxin CsTX-1 can greatly enhance *Drosophila* mortality [41]. These results suggest that the hyaluronidase and neuropeptide toxins found in HN-EVs could be considered as a way for spiders to increase the virulence of their venom.

### 2.6. HN-EVs Inhibit Viability of HUVECs

**Figure 6:**
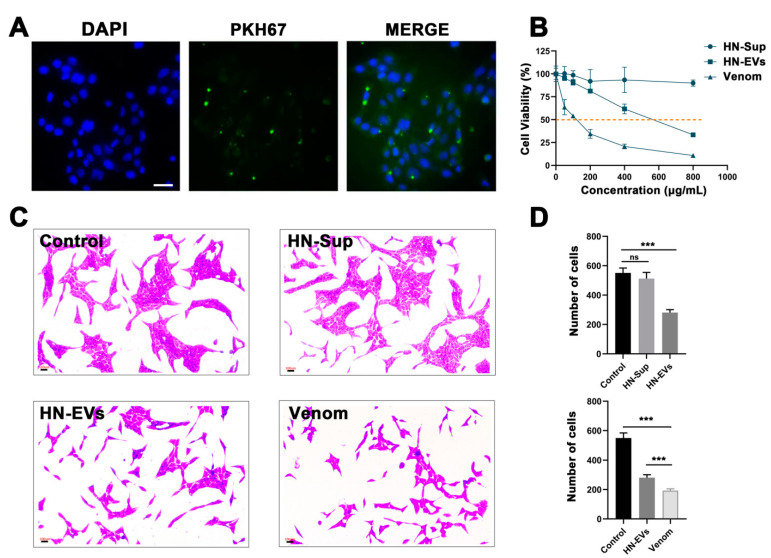
HN-EVs inhibit the viability of HUVECs. (**A**) Fluorescence microscopy analysis of PKH67-labeled HN-EV internalization by HUVECs. The green-labeled EVs were visible in the perinuclear region of recipient cells, scale bar: 50 μm. (**B**) HUVECs were treated with several concentrations of HN-EVs, HN-Sup, and whole venom, and cell viability was assessed by MTT assay. The half-inhibitory concentration (IC50) was determined to be 575 μg/mL (HN-EVs) and 100 μg/mL (whole venom). Results are shown as mean ± SD (*n* = 3). (**C**,**D**) Crystal violet staining was used to determine cell viability and count the number of cells in each treatment group. Results are shown as mean ± SD (*n* = 3). *** *p* < 0.001.

When spiders hunt and defend, their chelicerae puncture the epidermis of the target species, allowing the venom to quickly enter the subcutaneous tissues and disseminate through blood vessels to exert its effect. Therefore, to assess the influence of HN-EVs and whole venom on vascular endothelial cells, we used HUVECs to investigate the cellular functions of HN-EVs. First, fluorescence microscopy revealed that PKH67-labeled HN-EVs had been incorporated into HUVECs. After 8 h of incubation, green fluorescent dye PKH67-labeled HN-EVs were transferred to the perinuclear region of HUVECs. (Figure 6A). The half-inhibitory concentration (IC50) of HN-EVs and whole venom treatment on HUVECs was also determined. The IC50 for HN-EVs was 575 g/mL, and the IC50 for the whole venom was 100 g/mL (Figure 6B). Crystal violet staining analysis revealed that, when compared to the control group, an equal amount of HN-EVs and whole venom significantly inhibited HUVEC proliferation (Figure 6C). However, HN-EVs had a weaker inhibitory effect on HUVEC proliferation than whole venom (Figure 6D). The observed cytotoxicity difference between EVs and whole venom is not surprising. This result has also been observed in the study of snake venom EVs [22]. HN-EVs are a subset of the whole venom, and thus the whole venom may contain more cytolytic peptides with cytotoxic activity (Figure 3A). Our findings confirmed that HN-EVs are functional, but more research is needed to determine their specific activity.

### 2.7. The Influence of HN-EVs on the Cell Cytoskeleton and Cell Cycle of HUVECs

**Figure 7:**
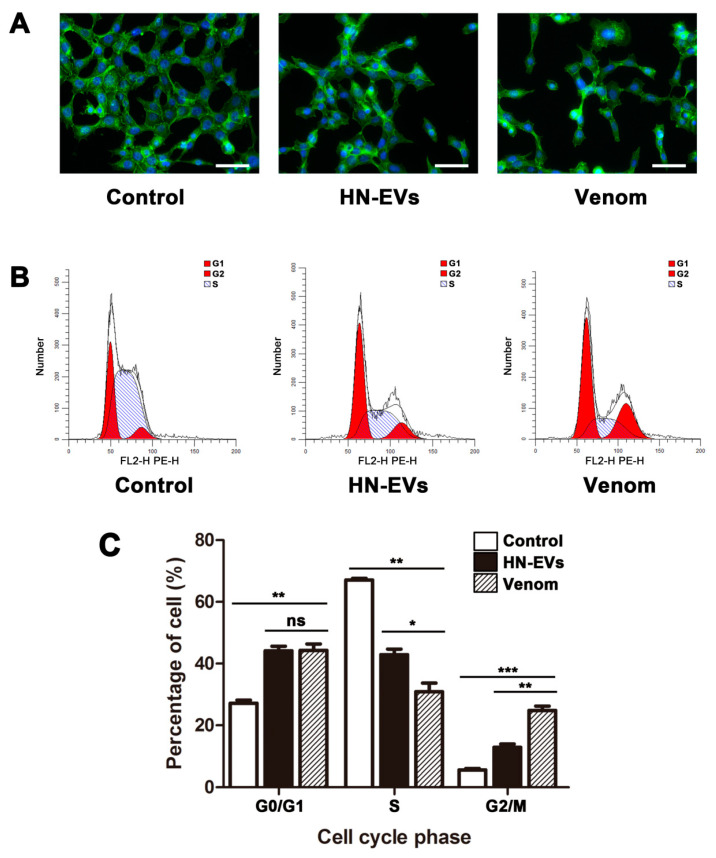
The influence of HN-EVs on the cell morphology and cell cycle of HUVECs. (**A**) Fluorescein-phalloidin staining was used to observe cell morphology and changes in actin filament organization (green) and nucleus (blue) of HUVECs. Scale bar: 50 μm. (**B**,**C**) The cell cycle distribution was detected using flow cytometric analysis and was quantified. Results are shown as mean ± SD (*n* = 3). No significance (ns). * *p* < 0.05, ** *p* < 0.01, *** *p* < 0.001.

To further investigate HN-EV effect on HUVECs, we examined the effects of HN-EVs on the actin organization and cell cycle of HUVECs. HUVECs exhibit disrupted fiber distribution, loss of intercellular contact, and decreased cell number per site after 48 h of exposure to HN-EVs and whole venom (Figure 7A). The cell cycle regulates cell proliferation. To determine whether the decrease in cell viability is due to cell cycle arrest, we examined the effects of HN-EVs on the cell cycle of HUVECs. The HN-EVs and whole venom treated cells exhibited G0/G1 and G2/M cell cycle arrest, as shown in Figure 7B,C by a significantly increased proportion of G0/G1 and G2/M cells (*p* < 0.01). These results suggest that HN-EVs may inhibit HUVEC growth by changing the cytoskeleton and arresting the cell cycle. It is worth noting that whole venom has a more substantial G2/M arresting effect than HN-EVs (*p* < 0.01). This result may also explain why whole venom has a more substantial proliferation inhibitory effect on HUVECs than HN-EVs (Figure 6B). Our research indicates that HN-EVs are biologically active, but their specific activities require further research.

## 3. Discussion

The characterization and functional analysis of EVs in snake and parasitic wasp venom further demonstrated that venom is a potent and diversified biochemical weapon [19,22]. This study isolated and characterized EVs from spider venom, revealing HN-EV protein composition, collagenase activity, and cellular functions. This study showed EVs in the venom of *Ornithoctonus hainana*, which could be an auxiliary method of venom secretion or a mechanism of mixed virulence factors in the envenomation process.

The two main pathways by which EVs are released from cells are via the formation of microvesicles and exosomes. Microvesicles are formed by plasma membrane vesiculation or blebbing [42]. Exosomes are released into the extracellular environment upon fusion of multi-vesicular bodies (MVBs) with the plasma membrane [28]. The apical portion of the glandular epithelium cells is rich in secretory vesicles, according to structural and ultrastructural descriptions of the venom glands of *Loxosceles intermedia* [23], *Cupiennius salei* [24], *Agelena labyrinthica* [25], *Vitalius dubius* [26], and *Argiope bruennichi* [27]. In this study, transmission electron microscopy revealed the MVB structure in the glandular epithelium cells of the *Ornithoctonus hainana* venom gland, as well as an exosome-like vesicle structure in the lumen. Interestingly, HN-EVs express the arthropod EV marker protein Tsp29Fb, which is homologous to the mammalian exosomal marker CD63 [14]. The mammalian exosomal marker HSP70 was also identified in HN-EVs through LC-MS/MS analysis [33]. These results indicate that the origin of HN-EVs seems to be consistent with exosomal biogenesis. However, our findings should be interpreted with caution, because while we show that HN-EVs can be produced via the exosomal biogenesis pathway, we do not rule out the possibility of other EV production pathways (plasma membrane vesiculation) in venom. In future studies, the origin of HN-EVs will be evaluated further using histochemical analysis.

Spiders use a combination of lethal venom and silk webs to subdue their prey while consuming the least amount of energy. These adaptations allow spiders to catch game that is up to seven times their body weight [43], an astonishing ratio for predators of all taxa. Therefore, spider venom attracts broad interest because of its biochemical and structural properties [2,44]. Research on spider toxins is currently focused on single components isolated from the venom; however, toxin molecules in nature do not function in isolation. The mixed-use of toxin molecules was discovered to be a strategy used by cursorial generalist spiders [45,46,47].

The secreted virus-like particles found in the venom of parasitic wasps [48], which contain a variety of virulence and immune-related factors, have been renamed mixed-strategy extracellular vesicles (MSEVs) or venosomes [19,20]. Similarly, HN-EVs also include some virulence-related factors, primarily a variety of toxin peptides and virulence-related proteins. The peptide toxins in HN-EVs are mainly neurotoxins. For example, HNTX-I can block rat sodium channels and insect sodium channels [49,50]. HNTX-VI inhibits the inactivation of voltage-gated sodium channels and reduces the peak sodium current of cockroach DUM neurons [51]. HNTX-IV.3 can selectively block neuronal tetrodotoxin-sensitive voltage-gated sodium channels [52]. HNTX-VII, HNTX-III-16, U3-TRTX-Hhn1r, and HNTX-XIV-7 are ion channel toxins that have the potential to block voltage-gated sodium channels [30,31,32]. Sodium channels are widely distributed in the neuron membranes of insects and mammals [52]. The combined function of these neurotoxins mixed in HN-EVs remains to be explored. However, in the *Cupiennius salei* spider venom, the weakly neurotoxic CSTX-13 enhances the paralytic activity of the neurotoxin CSTX-1 when it is administered with the latter at its entirely nontoxic physiological concentration [53,54]. This indicates that the mixed-peptide toxins in HN-EVs may also significantly affect target species. Moreover, we identified hyaluronidase in HN-EVs and verified their HA degradation activity. Hyaluronidase can promote the spread of virulence factors in spider venom [37]. The lethality of CSTX-1 mixed with hyaluronidase in venom after injection into flies is better than that of a single component [41]. This also implies that the mix of hyaluronidase and neurotoxins in HN-EVs may be advantageous to the envenomation process.

To assess the potential functions of HN-EVs, we investigated the effect of HN-EVs on HUVEC cells in vitro. The results indicate that HN-EVs can be taken up by HUVECs and inhibit cell activity. Furthermore, HN-EVs can cause morphological changes in HUVECs and cell cycle arrest. This function is similar to that of snake venom extracellular vesicles (SVEVs). SVEVs can disrupt hemostasis and cause endothelial cell degeneration, increasing the efficiency of prey paralysis [22]. Moreover, parasitoid wasp venom EV interactions with the blood cells of host larvae are linked to hematopoietic depletion, immune suppression, and parasite success [21]. These findings indicate that the functions of EVs in venom are varied and targeted.

In recent studies on drug delivery vehicles, EVs derived from various cells were recognized as the most promising drug delivery systems [55]. We hypothesize that the EVs secreted in the venom may act as a naive carrier of virulence factors for venomous animals. The flexible envelope structure of EVs in venom may make virulence factor release in the target species more stable and long-lasting. The different functional proteins and virulence factors in EVs make the envenomation process more targeted [21] and diverse [22]. Hence, we believe that EVs in spider venoms are not only an auxiliary venom secretion method but also a potential mixed strategy in the spider envenomation process. However, the biochemical and biological assays used in our study only confirmed that HN-EVs are functional. More research is needed to determine their specific activity in vivo and in vitro. While the Theraphosidae family provides a good model for studying EVs in spider venom, spider venoms are generally complex cocktails undergoing highly rapid evolution [56]. Hence, our future research will focus on characterizing and comparing EVs in venom from various spider families and attempting to explain the role of EVs in venom from the perspective of species evolution. This work will help define the function and significance of EVs in spider venom.

## 4. Conclusions

The present study isolates and characterizes HN-EVs for the first time, investigates the origin of HN-EVs, and establishes the presence of EVs in spider venom. The HN-EV ultrastructure and marker protein are consistent with those of typical extracellular vesicles, and the origin of HN-EVs is at least partially derived from exosomal biogenesis. Furthermore, 150 proteins were identified in HN-EVs. These proteins include virulence proteins such as neurotoxins and hyaluronidase, as well as vesicle transport proteins. The hyaluronidase activity of HN-EVs was confirmed. HN-EVs can inhibit HUVEC proliferation and cause morphological changes in HUVECs as well as cell cycle arrest. These study results shed new light on the envenomation process of spiders and serve as a valuable reference for the study of venom EVs.

## 5. Materials and Methods

### 5.1. Sample Preparation

Adult *Ornithoctonus hainana* were collected in China’s Hainan province and fed with mealworms and water once a week. For venom collection, approximately 60 spiders were used. The venom was harvested using an electrical stimulation methodology similar to the one described in our previous study [57]. The EVs in the venom were separated immediately after the spider venom was collected to prevent EV degradation. To observe the secretion of EVs, the spider venom claw (venom was not collected) was dissected in PBS to isolate the venom gland, which was fixed in 3.5% glutaraldehyde for TEM analysis.

### 5.2. Purification of EVs in Spider Venom

HN-EVs were isolated from spider venom using density gradient centrifugation as previously described (Figure 2A), with minor modifications [19,48]. Briefly, the venom was centrifuged at 10,000× *g* for 5 min to remove glandular endothelial cells and other cell debris. The supernatant was layered on preformed 10–50% (*w*/*v*) Nycodenz (Sigma Aldrich, St. Louis, MO, USA) gradients in PBS in 12.5 mL Ultra-Clear tubes. First, the tubes were centrifuged at 100,000× *g* for 120 min at 4 °C in an SW41Ti rotor in a Beckman L90K centrifuge (Beckman Coulter, Woerden, Netherlands). Then, the light-scattering band was washed with PBS and centrifuged at 100,000× *g* for 70 min. Finally, the pellet (which contained EVs) was resuspended in 100 μL PBS. The protein concentration was determined by the BCA protein assay kit (Thermo Fisher, Waltham, MA, USA). The EVs were either used right away or stored at −80 °C for later use.

### 5.3. Transmission Electron Microscopy

To observe the ultrastructure of glandular epithelial cells and HN-EVs, the venom gland was fixed in PBS with 3.5% glutaraldehyde for 24 h at 4 °C. After fixation in the same buffers with 2% osmium tetroxide, the samples were dehydrated in a graded ethanol series before being included in Epon and ultrafine sections. Uranyl acetate and lead citrate were used to stain the thin sections. TEM analysis was performed on the isolated HN-EVs as previously described [29]. Briefly, 20 μL of purified EVs was adsorbed onto copper grids for 1 min at room temperature before being adsorbed onto superfluous EVs and stained with 30 g/L phosphotungstic acid (pH 6.8) for 5 min at room temperature. They were then dried under a half-watt lamp. The thin sections and EVs were imaged using TEM (FEI, Hillsboro, OR, USA), and AMT CCD cameras (Advanced Microscopy Techniques, Danvers, MA, USA) were used to capture the images.

### 5.4. SDS-PAGE and Western Blotting Assay

The whole venom and HN-EVs were prepared in RIPA buffer (Beyotime, Shanghai, China) with a protease inhibitor cocktail (Selleck, Shanghai, China). Then, 20 μg/well of spider venom or HN-EVs was separated on a 12% polyacrylamide gel using SDS-PAGE (sodium dodecyl sulfate-polyacrylamide) gel electrophoresis and stained with Coomassie Brilliant Blue. For Triscine-PAGE analysis, the spider venom and purified HN-EVs were loaded onto a 16% Tricine gel and run at 120 V for 80 min. When the bromophenol blue reached the bottom of the gel, the whole venom and HN-EV protein was transferred to the PVDF membrane (Millipore, Billerica, MA, USA) for western blot analysis. The PVDF membrane was blocked in TBST (0.05 %) with 4% BSA solution for 2 h at room temperature and then incubated at 4 °C overnight with the primary antibody against arthropod EV Marker CD63 (Tsp29Fb) [14] (1:1000, Santa Cruz biotechnology, Santa Cruz, CA, USA). The blot was observed using the Chemi DocXRS imaging system (Bio Rad, Hercules, CA, USA) after incubation with a secondary antibody (1:2000, Abcam, Cambridge, UK) at room temperature for 1 h.

### 5.5. Sample Preparation and Digestion

HN-EVs were sonicated three times on ice using a high-intensity ultrasonic processor (Scientz, Ningbo, China) in lysis buffer (8 M urea, 1% Protease Inhibitor Cocktail). The remaining debris was removed by centrifugation at 12,000× *g* at 4 °C for 10 min. Finally, the supernatant was collected, and the protein concentration was determined with the BCA kit according to the manufacturer’s instructions. For digestion, the protein solution was reduced with 5 mM dithiothreitol for 30 min at 56 °C and alkylated with 11 mM iodoacetamide for 15 min at room temperature in darkness. The protein sample was then diluted by adding 100 mM TEAB to ensure a urea concentration less than 2M. Finally, trypsin was added at 1:50 trypsin-to-protein mass ratio for the first digestion overnight and 1:100 trypsin-to-protein mass ratio for a second 4 h digestion.

### 5.6. MS/MS Analysis of Venom HN-EV Proteins

The tryptic peptides were dissolved in 0.1% formic acid (Solvent A) and loaded directly onto a homemade reversed-phase analytical column (15 cm length, 75 μm i.d.). The gradient was comprised of an increase from 6% to 23% Solvent B (0.1% formic acid in 98% acetonitrile) over 26 min, 23% to 35% over 8 min, up to 80% over 3 min, and holding at 80% for the last 3 min, all at a constant flow rate of 400 nL/min on an EASY-nLC 1000 UPLC system. The peptides were subjected to an NSI source followed by tandem mass spectrometry (MS/MS) in Q Exactive™ Plus (Thermo Fisher, Waltham, MA, USA), coupled online to the UPLC. The electrospray voltage applied was 2.0 kV. The *m*/*z* scan range was 350 to 1800 for a full scan, and intact peptides were detected in the Orbitrap (Thermo Fisher, Waltham, MA, USA) at a resolution of 70,000. Peptides were then selected for MS/MS using an NCE setting of 28, and the fragments were detected in the Orbitrap at a resolution of 17,500. A data-dependent procedure was used that alternated between one MS scan followed by 20 MS/MS scans with 15.0 s dynamic exclusion. Automatic gain control (AGC) was set at 5E4. The fixed first mass was set as 100 *m*/*z*.

### 5.7. Database Search and Bioinformatic Analysis

To analyze the proteome of HN-EVs, the resulting MS/MS data were processed using the Maxquant search engine (v.1.5.2.8, Thermo Fisher, San Jose, CA, USA). Tandem mass spectra were searched against the UniProt database (Araneae) concatenated with a reverse decoy database. Trypsin/P was specified as the cleavage enzyme, allowing up to 4 missing cleavages. The mass tolerance for precursor ions was set as 20 ppm in the first search and 5 ppm in the main search, and the mass tolerance for fragment ions was set as 0.02 Da. Carbamidomethyl on Cys was specified as fixed modification, and acetylation modification and oxidation on Met were specified as variable modifications. The false discovery rate (FDR) was adjusted to <1%, and the minimum score for modified peptides was set to >40. The subcellular localization of the protein was analyzed by the Wolfpsort tool (http://www.genscript.com/psort/wolf_psort.html, 15 March 2021). General functions analysis was performed with the tools on the Swiss-Prot database (http://www.uniprot.org, 23 November 2019).

### 5.8. Hyaluronic Acid Hydrolysis Assay

To evaluate the hyaluronidase activity of HN-EVs, 10 μg of hyaluronic acid (HA) (Sigma Aldrich, St. Louis, MO, USA) was incubated overnight with 40 μg whole venom and HN-EVs in a final volume of 40 μL 0.2 M NH_4_Ac, 0.15 M NaCl buffer (pH 5.8) at 40 °C. Samples after incubation were loaded onto 1% agarose gels in 0.04 M Ba(Ac)_2_ (pH 5.8), and electrophoresis was performed at 50 mA in 0.05 M 1,2-diamino propane buffer (pH 9) for 3 h. After electrophoresis, the gel was fixed for 4 h in a 0.1% (*w*/*v*) cetyltrimethylammonium bromide solution, followed by 0.5% toluidine blue (Sangon, Shanghai, China) staining to reveal HA. Finally, the gel was further stained with Stains-All (Sangon, Shanghai, China); HA was bright blue after staining.

### 5.9. HN-EV Uptake Assay

To observe the uptake of HN-EVs by HUVECs, in accordance with the manufacturer’s recommendations, HN-EVs were labeled with a PKH67 green fluorescent cell Linker Kit (Sigma Aldrich, USA). The green fluorescently labeled EVs and HUVECs were incubated at 37 °C for 3 h. Then, the incubated cells were washed three times with PBS and fixed with 4% paraformaldehyde for 15 min. The fixed sample was washed with PBS three times and the nucleus was stained with DAPI (Invitrogen, Carlsbad, CA, USA). The fluorescence signal of the sample was analyzed with a fluorescence microscope (Leica DMI6000B, Wetzlar, Germany).

### 5.10. Cell Viability Assays

To evaluate the biological activity of HN-EVs, the MTT colorimetric method was used to determine the effect of HN-EVs on the cell viability of HUVECs. The HUVECs (0.5 × 10^4^ cells) were seeded in 96-well plates and cultured at 37 °C for at least 48 h before being treated with HN-EVs and whole venom. After the incubation, 1.2 mM MTT reagent (Sigma Aldrich, USA) was added, followed by DMSO 50% (*v*/*v*) after 4 h at 37 °C with 5% CO_2_. Each group’s concentration was six parallel holes, which were repeated three times. Formazan was quantified by measuring the absorbance at 570 nm referring to 630 nm wavelength. The variable slope curve-fitting using GraphPad Prism Software (GraphPad Software, San Diego, CA, USA) was used to assess IC50 data. The methanol was used to fix the cells for 15 min before straining them with a 0.5 % crystal violet solution for 7 min. ImageJ software (NIH, Bethesda, MD, USA) was used to analyze nine sites in each experiment in three replicates.

### 5.11. F-Actin Organizational Changes

To investigate the effects of exposure to HN-EVs on the actin cytoskeleton of HUVECs, TRITC-Phalloidin (Solarbio, Beijing, China) was used to stain F-actin according to the manufacturer’s instructions. Briefly, after 48 h of treatment, HUVECs were fixed in 4% paraformaldehyde, followed by permeabilization with 0.1% Triton X-100 and staining with 2 U/mL TRITC-Phalloidin for 30 min. Then, the nucleus was stained with DAPI (Invitrogen, Carlsbad, CA, USA). The fluorescence signal of the sample was analyzed with a fluorescence microscope (Leica DMI6000B, Wetzlar, Germany).

### 5.12. Cell Cycle Assay

To assess the impact of HN-EVs on the cell cycle of HUVECs, flow cytometry was used in all groups to determine the cell cycle. Briefly, HUVECs treated with the whole venom and HN-EVs for 48 h were trypsinized and carefully washed three times with PBS. HUVECs were fixed with ethanol at 4 °C for 24 h. Then, cells were washed twice in PBS containing 1 µg/mL RNase. Propidium iodide (PI) was added to a final concentration of 20 g/mL. All cells were transferred to 96-well plates, and FACS Array (Becton Dickinson, San Jose, CA, USA) was used to evaluate flow cytometry data. The strength of the PI signal was histogrammed, and the percentages of cells in the G1, S, and G2 phases were calculated.

## Figures and Tables

**Table 1:** Function classification of HN-EVs candidate proteins.

Classification	Accession	Gene	Description
Vesicular transport associated proteins	A0A4Y2J3E9	RAB11FIP3	Rab11 family-interacting protein 3
	A0A4Y2IWS4	HSP70B2_4	Heat shock protein 70 B2
	A0A4Y2H3W6	Vat1l_2	Synaptic vesicle membrane protein VAT-1-like
	A0A2P6KJS2	NCL1_37780	Golgin subfamily A member 4
	A0A4Y2J0H4	AVEN_242130_1	Putative antigen B membrane protein
*Ornithoctonushainana* peptide toxins	D2Y2C3	HNTX-VII	U5-theraphotoxin-Hhn1a
	D2Y2D7	HNTX-IV.3	Mu-theraphotoxin-Hhn1b 3
	D2Y2I6	HNTX-III-16	Mu-theraphotoxin-Hhn2i
	P0CH72	U3-TRTX-Hhn1r	U3-theraphotoxin-Hhn1r
	D2Y2E6	HNTX-XIV-7	U8-theraphotoxin-Hhn1f
	P0CH70	HNTX-VI	U1-theraphotoxin-Hhn1a
	D2Y1X8	HNTX-I	Mu-theraphotoxin-Hhn2b 3
Other spiders peptide toxins	P84835	GxTx1E	Kappa-theraphotoxin-Pg1a
	B1P1A6	JZTX-VIII	U3-theraphotoxin-Cg1a
	P0CH43	Tau-TRTX-Hs1a	Tau-theraphotoxin-Hs1a
	B1P1K1	JZTX-82	Secretory protein
	P84836	GxTX-1D	Kappa-theraphotoxin-Pg1b
	B1P1D8	JZTX-37	U13-theraphotoxin-Cg1b
	B1P1G5	JZTX-40	U21-theraphotoxin-Cg1c
	P62520	JZTX-III	Beta/kappa-theraphotoxin-Cg1a
Virulence associated proteins	A0A2L2YBK4	Hyal	Hyaluronidase
	A0A2P6KXQ8	NCL1_24342	Serine/threonine protein kinase-like
	A0A4Y2HPX8	AVEN_37080_1	Metallothionein-like protein 3B

## Data Availability

Not applicable.

## Supplementary Materials

The following are available online at https://www.mdpi.com/article/10.3390/toxins13080579/s1, Table S1: a total of 150 non-redundant proteins in HN-EVs; Table S2: identification of total peptides in HN-EVs by LC-MS/MS.
